# Representation of Women in Top Executive Positions in General Medical-Surgical Hospitals in the United States

**DOI:** 10.1089/whr.2020.0111

**Published:** 2021-05-11

**Authors:** Jason N. Mose

**Affiliations:** Department of Health Services and Information Management, East Carolina University, Greenville, North Carolina, USA.

**Keywords:** hospital leadership, gender diversity in hospital leadership, gender disparities

## Abstract

***Background:*** Earlier surveys have reported a lack of representation of women in hospital leadership positions. This study sought to investigate the proportion of women in senior hospital leadership positions and to investigate whether hospital and community factors are associated with the likelihood of having women in executive positions.

***Methods:*** The main data of 4397 hospitals were sourced from the American Hospital Association. The study calculated the proportion of women for each position, with county-level adjusted standard errors and logistic transformed confidence intervals to determine the variation of women in chief officer positions across hospitals. The study also used multilevel mixed-effects logistic regressions to estimate the probability of having a female chief officer.

***Results:*** Women executives generally were underrepresented in all positions except in chief human resources and chief nursing officer positions, 73% (95% confidence interval [CI] 7175) and 91% (95% CI 8694), respectively. Women accounted for 13% (95% CI 821) of system chief executive officers (CEOs) and only 27% (95% CI 2628) of hospital CEOs. System size (number of hospitals) and hospital size (beds capacity) variables were statistically significant in five of the eight positions investigated. Overall, women were less likely to hold chief positions in large hospitals (400 beds and over) compared to small hospitals (under 100 beds).

***Conclusion:*** Women were underrepresented in hospital top positions and were less likely to hold executive positions in large systems or hospitals. The results suggest structural barriers that hinder women from advancing to top hospital positions.

## Introduction

Data from the Bureau of Economic Statistics show that in 2018, 79% of the health services workforce were women and 75% of employees in hospitals were women.^1^ Further, data from the Equal Employment Opportunity Commission show that women account for 72% of first or mid-level managerial positions and executive or senior administrative levels in the sector.^2^ Over three million professionals worked in the health care and social assistance private sector in 2018, of whom 80% were women.^2^ However, national surveys of the chief officer positions also referred to as C-suite, report persistent gender disparities. For example, a 2012 survey revealed that given the same amount of time of experience as men in health care management, only 11% of women achieved chief executive officer (CEO) positions compared to 22% of men.^3^ American College of Healthcare Executives periodic surveys also show not much has changed since 1990 in terms of the proportion of women ascending to the CEO position. For example, in 1990, 11% of women rose to CEO positions; 8% in 1995; 11% in 2000; and 12% in 2006.^35^

Although the benefits of leadership diversity are well documented in various industries, gender diversity has been a concern in corporate and health care industry in the United States and across other advanced economies like Canada and Great Britain.^610^ Studies have noted that socially diverse teams tend to be more innovative. The right kind of leadership can lead to breaking down barriers, enhancing team effectiveness, and activation of resilience in times of a crisis.^1115^ Also, political science literature indicates that when women are represented in legislative bodies, discussion and implementation of women policy priorities also tend to increase.^16,17^ It follows then that similar benefits might be achieved if hospital leadership reflect the gender diverse workforce and communities hospital leadership serve. This is especially important given the complexity of health care service delivery in an acute care setting. In any given day, managing an acute care hospital is a complicated endeavor, but the pressure is much higher during a major crisis, such as the coronavirus disease 2019 (COVID-19) pandemic or a natural disaster. For example, in a such a crisis, a hospital leader's foremost priority is the health and safety of the health care workers, without whom the hospital will not survive or function to serve the community.^18^ To achieve this goal, a leader must reach into both tangible resources such as financial and infrastructure, and intangible such as relational resources.^15^ The hospital leader also must understand the needs and circumstances of employees who themselves are diverse in all of the traditional measures, but also are affected by widely varying life, work, and family conditions.^18^

Leadership diversity, including gender diversity, therefore, is of two critical ingredients that hospitals need to operate successfully. Despite the known benefits and the moral argument of not sidelining representation of more than half of the hospital workforce, previous research indicates a dearth of women leaders in top positions. Several reasons account for the dismal representation of women in top leadership ranks. For example, in 1995, the National Glass Ceiling Commission identified three categories of barriers to women ascending to CEO positions.^19^ These included societal, internal, and government barriers. Lack of educational opportunities, prejudice, and bias related to the intersectionality of gender, race, and ethnicity, recruitment practices, pipeline barriers that restrict advancement, lack of mentoring, and lack of government monitoring and enforcement contribute to constraining women from advancing to top positions.^19^

Paradoxically, since 1990s, progress has been made in several of the barriers identified in the Glass Ceiling Commission report; and yet, the gender disparities have persisted. Take for example the education and pipeline issue. The number of women enrolling and graduating from programs that feed into health care management has grown to almost parity or more than men. The Association of American Medical Colleges (AAMC) data show that since the 1995 academic year, over 40% of graduating medical doctors are women. In 2015, 39% of full-time faculty in medical schools were women.^20^ However, of the 2768 permanent deans of AAMC member institutions, only 437(16%) were women.^20^ Data from other training programs that feed the health care managerial and administrative ranks also show a higher proportion of women graduating since the early 1990s. For example, in the 20092010 academic year, of the 6323 health, health care administration, or management master's degrees conferred, 4378 (69%) were awarded to women.^21^ In the same year, 46% of those graduating with a Master of Business Administration, management, marketing, and other related areas were women.^21^ In some other specialization categories, such as, accounting and finance, a majority (64%) of the degrees awarded were to women.^21^ Thus, evidence suggests that the pipeline is not the only issue that explains the dearth of women in top hospital leadership positions.

Therefore, the purpose of this study is twofold: first, assess the proportion of women in the C-suite; second, investigate whether hospitals' organizational structures and community factors are associated with the likelihood of having women in top leadership positions in general medical-surgical hospitals in the United States.

## Methods

In this cross-sectional study, datasets from the following entities were used: American Hospital Association (AHA), Centers for Medicare and Medicaid Services (CMS), U.S. Census Bureau, and other supplemental datasets. Data on eight top officer positions were sourced from the AHA data covering the calendar year 2017. The positions include hospital system administrator, CEO/hospital administrator, chief financial officer (CFO), chief human resources officer (CHRO), chief information officer (CIO), chief medical officer (CMO), chief nursing officer (CNO), and chief operating officer (COO). Other variables taken from the AHA dataset include a system hospital indicator (whether a hospital has set up an accountable care organization [ACO]) and hospital bed capacity. Other variables were extracted from the CMS provider of service file, the impact file, and the hospital service area file. Community factors such as county characteristics and area disadvantage were sourced from the American Community Survey (20142018).^22^

The hospital characteristics such as hospital ownership, teaching status, rurality, safety-net status, and bed capacity were adjusted for the system administrator position. The adjustment stems from a fact that a health care system, for example, can have hospitals located both in rural and urban areas, and own small, medium, or large hospitals among other characteristics. Another complication in classifying a system is the complexity of ownership status. For instance, some for-profit systems also own or manage not-for-profit hospitals. This study took several steps to ensure a reasonably accurate reflection of the system characteristics; first, by enumerating the number of hospitals per system and, second, by designating the system to a specific characteristic if the majority (50% or more) of its member hospitals belonged to such characteristics. For example, if more than 50% of hospitals under the system were for-profit, the system was categorized for profit. The same methodology was applied for rurality, bed capacity, and safety-net status variables. It appears there was no overlap on teaching hospital status. Therefore, if a system had a major teaching hospital, minor teaching, and nonteaching, the entire system was categorized as a teaching hospital system.

The social titles, such as Mr., Mrs., or Ms., of the hospital leaders were used to identify their gender. However, in some cases, the professional title of the individual was recorded instead of the pronoun. In such cases, the Gender Application Programming Interface (API) application was used to identify the gender of the leader.^23^ Leaders with the professional title instead of a pronoun varied from position to position. They ranged from less than 1% in the CFO position to about 26% in system administrator position and 98% in CMO position. Ten names missing the pronouns were manually searched to ascertain the accuracy of the Gender API, and the application was accurate in each of those cases.

The study focus is limited to general medical-surgical hospitals in the 50 states and the District of Colombia. Some hospitals did not report some positions, and therefore, the number of hospitals included varied from position to position. For example, 4397 hospitals reported a CEO position, while only 1544 hospitals reported having a COO position.

The study computed the proportion of women for each position; with county-level adjusted standard errors and logistic transformed confidence intervals to determine the variation of women in chief officer positions across hospitals. The study used multilevel mixed-effects logistic regressions to estimate the probability of having a female chief officer. The method was chosen because of the nested nature of the data and the idea that hospital hiring decisions might be influenced by county and regional level cultural and social-economic factors. For all positions, except the COO, the study fitted three-level random-intercept models with county-level nested in the census division level. Due to convergence issues, even after several tries to obtain better starting values, a logit model was fitted for the COO position. Finally, the study reported computed marginal effects.

All analyses were conducted using Stata 15.1.

## Results

**FIG. 1.**
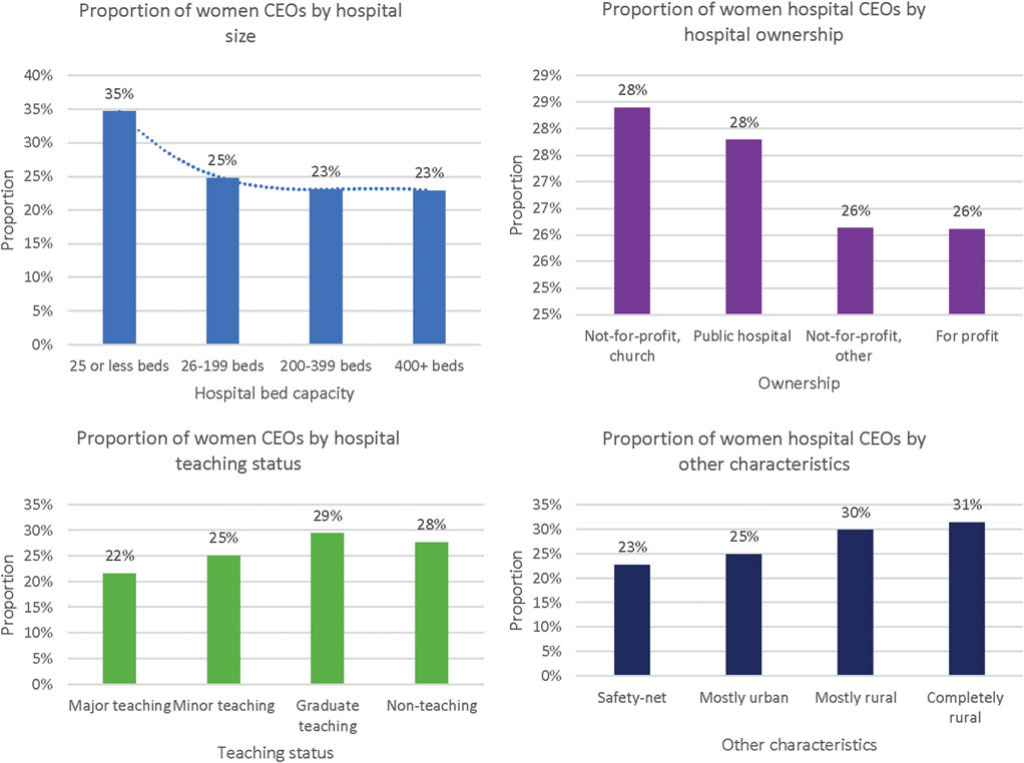
Proportion of women CEOs by hospital characteristics. CEOs, chief executive officers.

Table 1 reports the proportions of women leaders in each reviewed position. The number of hospitals reporting varied across positions. For example, all (4397) general hospitals included in the study reported a CEO position also called a hospital administrator. Fewer hospitals reported having other positions such as CFO (3590), CNO (3003), and COO (1544). Women executives generally were underrepresented in most positions except the chief human resources and CNO positions, 73% (95% CI 7175) and 91% (95% CI 8694), respectively. For example, women accounted for 13% (95% CI 821) of system CEO, and only 27% (95% CI 2628) of hospital CEO (Table 1). Figure 1 reports the proportion of women CEOs by hospital characteristics, including the hospital size (number of beds), ownership, teaching status, and other factors such as location rural or urban. The results show that while women accounted for a minority share of CEO positions across hospital characteristics, small hospitals (25 beds or less), church-owned hospitals, graduate teaching, and nonteaching, completely rural, and mostly rural hospitals had a higher proportion of women as CEOs.

**Table 1.** Proportions of Women in a Chief Officer Position in General Medical-Surgical Hospitals

	Proportion (%)	Standard error	Logit	*N* (M/F)	Total *N*
			(95% confidence interval)		
System leader					
Male	87	0.02909	7992	2510	2891
Female	13	0.02909	821	381	
Hospital administrator/CEO					
Male	73	0.00393	7274	3220	4397
Female	27	0.00393	2628	1177	
CFO					
Male	65	0.01774	6169	2339	3590
Female	35	0.01774	3139	1251	
Chief human resource officer					
Male	27	0.00837	2529	945	3510
Female	73	0.00837	7175	2565	
CIO					
Male	72	0.00915	7074	2040	2844
Female	28	0.00915	2630	804	
CMO					
Male	86	0.02710	7891	2789	3256
Female	14	0.02710	922	467	
CNO					
Male	9	0.01733	614	281	3003
Female	91	0.01733	8694	2722	
COO					
Male	61	0.09290	3979	935	1544
Female	39	0.09290	2161	609	

CEO, chief executive officer; CFO, chief financial officer; CIO, chief information officer; CMO, chief medical officer; CNO, chief nursing officer; COO, chief operating officer.

Table 2 reports mixed-effects logistic regression results on the probability of having a female chief officer, ranging from system administrator to COO. Of the eight positions investigated, system size (number of hospitals) or hospital size (bed capacity) variables were statistically significant in five positions, suggesting that women are likely to lead small systems or smaller hospitals than larger systems or hospitals. For example, being a woman is associated with 8.3 (*p*<0.05) percentage points decrease in the probability of leading a system with 1845 hospitals compared to a system with 16 hospitals, all else held constant. Similarly, being female is associated with 6.7 (*p*<0.01) percentage points decrease in the probability of leading a large hospital (400+ beds) compared to a small hospital. Also, women are less likely to hold other chief positions in large hospitals; such positions as CHRO, 15.6 (*p*<0.01), and COO, 25.1 (*p*<0.01), percentage points, respectively.

**Table 2.** Probability of a Hospital Having a Female Chief Officer (Complete Case Analysis)

	System administrator	Hospital CEO	Hospital CFO	Hospital CHR	Hospital CIO	Hospital CMO	Hospital CNO	Hospital COO
Public hospital		0.006 (0.286)	0.023 (1.178)	0.019 (1.079)	0.024 (1.051)	0.008 (0.600)	0.002 (0.243)	0.094^***^ (2.881)
Public hospital system	0.081 (1.566)							
For-profit		0.004 (0.173)	0.005 (0.168)	0.018 (0.858)	0.035 (1.054)	0.007 (0.385)	0.011 (1.592)	0.030 (0.839)
For-profit system	0.144^**^ (2.194)							
Not-for-profit, church		0.028 (1.159)	0.047 (1.619)	0.026 (0.771)	0.044^*^ (1.771)	0.005 (0.292)	0.001 (0.172)	0.026 (0.666)
Not-for-profit church system	0.092^***^ (3.055)							
Completely rural		0.033 (0.911)	0.097^***^ (3.003)	0.057^*^ (1.766)	0.046 (0.695)	0.027 (0.665)	0.005 (0.297)	0.035 (0.637)
Completely rural system	0.077^**^ (1.991)							
Mostly rural		0.025 (1.157)	0.026 (0.850)	0.017 (1.076)	0.023 (0.751)	0.004 (0.172)	0.009 (1.049)	0.023 (0.672)
Mostly rural system	0.046 (1.486)							
Major teaching		0.013 (0.602)	0.034 (0.754)	0.024 (0.809)	0.052 (1.634)	0.012 (0.504)	0.006 (0.511)	0.001 (0.023)
Major teaching system	0.007 (0.701)							
Minor teaching		0.002 (0.102)	0.017 (0.431)	0.007 (0.735)	0.010 (0.395)	0.002 (0.149)	0.006 (0.825)	0.026 (0.857)
Minor teaching system	0.001 (0.035)							
ACO	0.053 (1.273)	0.030^*^ (1.878)	0.028^**^ (1.974)	0.015 (0.778)	0.033^*^ (1.826)	0.015 (0.769)	0.002 (0.425)	0.066^**^ (2.443)
Safety net		0.028 (1.063)	0.003 (0.093)	0.006 (0.175)	0.032 (1.108)	0.031 (1.117)	0.004 (0.423)	0.046 (1.320)
Safety-net system	0.099^***^ (2.823)							
16 hospitals system		0.013 (0.832)	0.021 (0.561)	0.041^***^ (2.656)	0.016 (0.554)	0.033 (1.497)	0.003 (0.278)	0.053 (1.432)
717 hospitals system	0.012 (0.571)	0.025 (0.940)	0.003 (0.098)	0.017 (1.411)	0.008 (0.301)	0.001 (0.039)	0.013 (1.398)	0.035 (0.949)
1845 hospitals system	0.083^**^ (2.153)	0.031^**^ (2.192)	0.052^*^ (1.889)	0.004 (0.121)	0.027 (1.015)	0.031^***^ (2.772)	0.001 (0.181)	0.021 (0.580)
50148 hospitals system	0.004 (0.110)	0.006 (0.271)	0.014 (0.471)	0.018 (0.518)	0.040 (1.379)	0.013 (0.693)	0.002 (0.402)	0.030 (0.759)
Medium hospital (26199 beds)		0.088^***^ (6.357)	0.106^***^ (4.374)	0.193^***^ (7.719)	0.029 (1.217)	0.003 (0.105)	0.012 (1.177)	0.100^***^ (2.671)
Medium hospital system	0.026 (1.207)							
Medium/large hospital (200399 beds		0.089^***^ (4.174)	0.110^***^ (3.363)	0.189^***^ (8.979)	0.008 (0.209)	0.031 (1.223)	0.010 (0.829)	0.160^***^ (3.284)
Medium/large hospital system	0.064^*^ (1.827)							
Large hospital (400+ beds)		0.067^***^ (2.836)	0.054 (1.175)	0.156^***^ (5.318)	0.039 (1.092)	0.022 (0.795)	0.017 (1.300)	0.251^***^ (4.190)
Large hospital system	0.151^**^ (2.009)							
Saidin Index	0.001 (0.680)	0.003^*^ (1.886)	0.001 (0.361)	0.001 (0.517)	0.005^***^ (2.672)	0.000 (0.159)	0.001 (1.308)	0.006^***^ (2.918)
HHI	0.030 (1.326)	0.004 (0.105)	0.050^*^ (1.806)	0.011 (0.210)	0.032 (0.531)	0.009 (0.267)	0.008 (0.710)	0.160^***^ (3.342)
Most disadvantaged	0.021 (0.856)	0.063^***^ (2.616)	0.078 (1.623)	0.047 (0.830)	0.114 (1.021)	0.010 (0.492)	0.005 (0.144)	0.037 (0.346)
Second most disadvantaged	0.002 (0.023)	0.019 (0.679)	0.009 (0.305)	0.004 (0.109)	0.074 (1.121)	0.009 (0.194)	0.001 (0.183)	0.053 (0.653)
Third most disadvantaged	0.120^**^ (2.266)	0.004 (0.155)	0.085^***^ (3.928)	0.057^*^ (1.906)	0.003 (0.056)	0.016 (0.339)	0.012 (0.565)	0.017 (0.273)
Dependency ratio	0.002^**^ (2.157)	0.002 (1.285)	0.001^*^ (1.663)	0.000 (0.177)	0.000 (0.439)	0.001 (0.945)	0.001 (0.869)	0.003 (1.489)
15 minutes commute	0.002^***^ (3.128)	0.000 (0.420)	0.001 (1.134)	0.001^*^ (1.840)	0.001 (0.830)	0.000 (0.505)	0.000 (1.105)	0.001 (0.491)
Public transport commute %	0.001 (1.153)	0.002^**^ (2.233)	0.001 (0.868)	0.000 (0.449)	0.002 (0.956)	0.002 (1.005)	0.002^*^ (1.900)	0.012^***^ (2.919)
African American %	0.002^**^ (2.421)	0.000 (0.274)	0.001 (1.126)	0.001^*^ (1.810)	0.000 (0.108)	0.001 (1.202)	0.000 (0.345)	0.000 (0.038)
Asian American %	0.003 (1.422)	0.004 (1.267)	0.006^***^ (4.376)	0.007^***^ (3.538)	0.009^*^ (1.838)	0.002^*^ (1.664)	0.000 (0.162)	0.041^**^ (2.413)
Hispanic %	0.001 (0.758)	0.000 (0.566)	0.001 (0.794)	0.002^***^ (3.203)	0.002^*^ (1.726)	0.002 (1.327)	0.000 (0.138)	0.010^***^ (4.230)
Foreign-born %	0.004^*^ (1.675)	0.003^*^ (1.850)	0.005^***^ (2.645)	0.002 (1.283)	0.005 (1.089)	0.002 (0.764)	0.003^*^ (1.787)	0.005 (0.643)
Uninsured rate	0.012^***^ (3.291)	0.001 (0.271)	0.001 (0.349)	0.005 (1.296)	0.003 (0.491)	0.010 (1.451)	0.002 (0.949)	0.006 (1.045)
Northeast	0.061 (1.593)	0.018 (0.559)	0.033 (1.017)	0.060^*^ (1.791)	0.100^*^ (1.841)	0.056 (1.178)	0.000 (0.009)	0.540^***^ (9.159)
Midwest	0.003 (0.094)	0.006 (0.477)	0.066^*^ (1.717)	0.026 (1.345)	0.021 (0.557)	0.034 (0.750)	0.040 (1.393)	0.187^***^ (4.368)
South	0.103^***^ (3.745)	0.001 (0.048)	0.071^***^ (2.893)	0.046^*^ (1.929)	0.027 (0.573)	0.023 (0.718)	0.007 (0.310)	0.137^***^ (3.193)
Small hospital system	0.007 (0.159)							
Observations	2847	4309	3527	3442	2790	3194	2953	1509

*t* statistics in parentheses.

^*^ *p*<0.1, ^**^p<0.05, ^***^p<0.01.

ACO, accountable care organization; HHI, Herfindahl-Hirschman Index.

The results suggest that women, when they are appointed system or hospital administrators, are more likely to be in a small system or a small hospital instead of larger systems or larger hospitals. Ownership is also statistically significant as an explanatory factor for the likelihood of a woman leading a hospital system. For example, on the one hand, being a woman is associated with 14.4 (*p*<0.05) percentage points decrease in the probability of leading a for-profit hospital system compared to other ownership types. On the other hand, being a woman is associated with 9.2 (*p*<0.01) percentage points increase in the probability of leading a church-owned not-for-profit hospital system. Other factors that were statistically significantly negatively or positively associated with some woman chief officer position included the Saidin Index, the Herfindahl-Hirschman Index (HHI), the county area disadvantage, racial/ethnicity of the county where the hospital is located, and geographic region, among other factors (Table 2).

## Discussion

The study confirms an early survey that found women underrepresented in the C-suite. However, the study adds more insight, including the disparity of women representation in large systems and hospitals. For example, the study results reveal that except for two senior hospital positions, CHRO and CNO, women are underrepresented in every other position. However, even in positions that have a high representation of women, they are less likely to work in a hospital with 26199 beds [19.3 (*p*<0.01) percentage points less likely], 200399 beds [18.9 (*p*<0.01) percentage points less likely], and 400 or more beds [15.6 (*p*<0.01) percentage points less likely]. The suggestion from the results is that even when women are a majority in a specific position such as CHRO, there are still barriers for them to rise to the same position in larger hospitals. While there is no clear evidence why women might serve more in some positions than others, in small systems and hospitals and not in larger ones, accumulated evidence suggests probable structural barriers.

First, the ACHE surveys going back to 1990 have consistently revealed that women tend to be in middle-level positions that are more specialized than men. For example, in 2012, 12% of women surveyed were involved in nursing leadership compared to 3% of men, and 13% versus 10% in planning, marketing, quality assurance, and other specializations remained unchanged since 2006.^3,5^ The survey reported that a higher proportion of men, 62%, were in general management compared to 50% of women; although, both women and men had similar educational training and women had more previous clinical experience than men.^3,24^ A similar finding in the corporate world provides insight into how entrenched the issue is across the industry. For example, an analysis of the biggest publicly traded firms found out that men on the way to C-suite overwhelmingly get the management jobs, in which a company's profits and losses hang in the balance.^25^ In contrast, women end up in positions in areas such as human resources, legal, or administration, critical functions that do not have profit-generating responsibility and are rarely a path to running a company.^25^ In the hospital sector, the implication is that women end up working in specialized fields that do not lead to positions such as COOs, which are stepping stones to the CEO seat. However, even when women end up in the COO position, internal mobility disparity persists; 28% of men who begin as COOs or senior vice presidents or associated administrators become CEOs compared to 22% of women who begin in the same position.^3^ Altogether, including the results from this study, there is strong evidence that intractably disadvantage women in rising up to the top positions.

Second, a 2019 corporate America *Women in the Workplace* study by McKinsey & Company and LeanIn.Org found that while progress has been made, women, more so women of color, are underrepresented at every level.^26^ One finding from the study that might explain this study's results and hospital sector's dearth of women in executive positions in general is what the authors refer to as the broken rung problem. The idea that the biggest hurdle for women to advance to a higher administrative role is the first step up to the manager position.^26^ The authors' finding is consistent with the evidence that shows that there are different inflection points for women hospital leaders based on functional areas. For the health care management track, the inflection point happens early in their careers, while those in clinical or administrative roles, the inflection point happens later in their careers.^27^ The inflection points were found to be related to education and training, experience, career management, family, networking, and mentorship and sponsorship.^27^ However, the authors report that two inflection points are shared among all the executives; education (completing a graduate degree) and experience (as a COO).^27^ The results reported in Table 2, the broken rung, and the inflection points evidence, taken together, appear to support the idea that women face challenges early on, especially in large organizations, which regrettably sets the rest of their career trajectories. Based on the reasons mentioned above, it is possible that women who advance are likely to do so in smaller organizations, where their talent is probably visible and more readily rewarded with a promotion.

The other interesting finding from this study concerned the two clinical leadership positions, CMO and CNO. First, it is surprising that not all 4397 hospitals included in the study had the positions (CMO *n*=3256 and CNO *n*=3003) or, at least, those hospitals with 25 beds or more. Second, in both cases, there was no statistically significant variable that explained the variation of women in those positions. Third, both positions had a different proportion of women, CMO 14% (95% CI 922), and CNO 91% (95% CI 8694). It is possible in both cases that variables that would have explained the variation were not included in the study, such as institutional culture. Based on earlier research on women in medicine, the women may face similar challenges to advance to CMO positions as those other counterparts in medicine such as the dean or chair of department.^6,8,9,20,28^ It is also possible that some women in both positions face age-old biases that equate leadership to masculinity and therefore hinder them from advancing.^29,30^

In the ACHE studies, the COO position was found to be one of the feeder positions to the CEO seat.^3,5^ The COO position regression results reveal, in contrast to the results from CMO and CNO positions, that several factors are statistically significantly associated with reaching the position. The hospital size, geographical region, market concentration (HHI), ACO status, and whether a hospital is a public hospital, among other factors were statistically significant. For example, a female in a hospital located in the Northeast region was associated with a 54% (*p*<0.01) decrease in the likelihood of being a COO compared to a hospital located in the West region. The female leader in a hospital located in the Midwest and South regions were, respectively, associated with 18.7 and 13.7 (*p*<0.01) percentage points decrease in the probability of being a COO. The only statistically significant measure that was positively associated with the likelihood of having a female COO was the HHI. A one-point increase in HHI was associated with a 16 (*p*<0.01) percent increase in the likelihood of a hospital having a female COO. These factors, some of which transcend hospital walls, larger hospital size, region, and ACO status, suggest substantial cultural and structural factors that probably contribute to limit the ascendance of women to the C-suite.

## Conclusion

The implications of the findings of this study are twofold. First, it appears that hospitals interested in encouraging gender diversity might need to investigate identification, retention and promotion practices, organization culture, and any other factor that shut out qualified women, especially in larger hospitals and systems. Second, hospitals must invest in interventions that have shown promise to improve the recruitment, retention, and promotion of women. Several articles have highlighted some interventions such as mentorship and leadership training programs that have shown the potential to lift some women to higher positions in the hospital sector.^3133^ However, given the slow progress, it appears that a single approach might not be a recipe for success. New interventions and new approach such as sponsorship must be necessary, in addition to other cultural and supportive environments for women.^27,30,3438^ Unless multipronged and concerted efforts, the top hospital leadership will continue shutting out qualified women and will lack gender diversity that is reflected in the hospital workforce.

The study had several limitations. First, the study is a cross-sectional investigation; therefore, it captures the associations at the point in time and not trends over time, especially given the high turnover rate in some positions such as CEO.^39^ However, the study also reviewed other studies such as those that survey hospital CEOs for more insights, and the results were consistent with those earlier surveys. The gender identification was restricted to the pronouns reported or those generated through the Gender API applications. These might not be accurate or a true reflection of the individual leader's gender identification. Finally, while careful consideration was applied on the selection of variables and the analytic approach, it is possible that other variables that are not readily available, such as institutional culture, leadership attitudes toward female leaders, and a different analytical method, might explain the variation in female representation in a chief officer position.

## Abbreviations Used

AAMC: Association of American Medical Colleges
ACO: accountable care organization
AHA: American Hospital Association
API: Application Programming Interface
CEO: chief executive officer
CFO: chief financial officer
CHRO: chief human resources officer
CI: confidence interval
CIO: chief information officer
CMO: chief medical officer
CMS: Centers for Medicare and Medicaid Services
CNO: chief nursing officer
COO: chief operating officer
COVID-19: coronavirus disease 2019
HHI: Herfindahl-Hirschman Index
